# Genotypic and clinical characteristics of long-term surviving adult patients with urea cycle disorders in Japan

**DOI:** 10.1186/s13023-026-04524-x

**Published:** 2026-07-31

**Authors:** Jun Kido, Keishin Sugawara, Naomi Yano, Kimitoshi Nakamura

**Affiliations:** 1 Department of Pediatrics, Faculty of Life Sciences, Kumamoto University, 1–1–1 Honjo, Chuo-ku, Kumamoto, 860–8556 Japan; 2 Department of Pediatrics, Kumamoto University Hospital, Kumamoto, Japan

**Keywords:** Genotype–phenotype correlation, Late onset, Long-term survivors, Pathogenic variants, Urea cycle disorders

## Abstract

**Background:**

Urea cycle disorders (UCDs) are rare inherited metabolic conditions characterized by impaired nitrogen disposal due to defects in enzymes or transporters. Although genotype–phenotype correlations have been investigated in patients with UCDs, the genetic characteristics and long-term clinical outcomes of adult survivors remain poorly understood. This study aimed to investigate the genotypic and clinical characteristics of adult long-term survivors with UCDs in Japan and to evaluate genotype–phenotype correlations.

**Methods:**

We conducted a nationwide survey and collected clinical and genetic data from 116 adult patients with UCDs. Pathogenic variants were identified in 48 patients, and clinical data including age at onset, peak plasma ammonia levels, intellectual outcomes, and long-term prognosis were analyzed. Particular attention was paid to genotype–phenotype correlations in male patients with ornithine transcarbamylase deficiency (OTCD).

**Results:**

Among 48 patients with identified variants (34 OTCD, 2 CPS1D, 6 ASS1D, 3 ASLD, 1 ARG1D, and 2 HHH syndrome), most OTCD variants in males were associated with late-onset phenotypes. Intellectual disability was present in 43.2% (19/44) of patients and was significantly associated with peak ammonia levels exceeding 360 μmol/L (*p* = 0.046), particularly in male OTCD patients (*p* = 0.018). Clinical presentations varied even among patients with identical variants.

**Conclusion:**

This is the first study to characterize the genetic and clinical profiles of adult long-term survivors with UCDs in Japan. These findings suggest that genotype information may help predict disease onset and clinical severity, although genotype alone is insufficient to fully predict long-term clinical outcomes.

**Supplementary Information:**

The online version contains supplementary material available at https://doi.org/10.1186/s13023-026-04524-x.

## Introduction

Urea cycle disorders (UCDs) are a group of rare inherited metabolic diseases characterized by impaired detoxification of nitrogen due to deficiencies in specific enzymes or transporters of the urea cycle. This cycle, essential for the conversion of toxic ammonia into urea for excretion, involves six key enzymes—carbamoyl phosphate synthetase 1 (CPS1), N-acetylglutamate synthase (NAGS), ornithine transcarbamylase (OTC), argininosuccinate synthetase 1 (ASS1), argininosuccinate lyase (ASL), and arginase 1 (ARG1)—along with two mitochondrial transporters, ornithine transporter 1 (ORNT1) and citrin [1].

Complete loss of function in any of these proteins typically results in neonatal-onset hyperammonemia, which presents within the first few days of life with acute and life-threatening symptoms, including lethargy, seizures, poor feeding, abnormal respiration, and coma [2, 3]. By contrast, partial enzyme deficiencies often remain asymptomatic until later in life and may manifest during catabolic stress—such as infections, surgery, or childbirth—as late-onset UCDs [4]. These forms are generally associated with milder hyperammonemia and nonspecific symptoms such as vomiting, fatigue, and behavioral changes. Neuropsychiatric symptoms—including delusions, hallucinations, and psychosis—may also occur, particularly in adults with late-onset disease [1, 5].

Increasing evidence indicates that residual enzymatic activity is a key determinant of disease severity. Variants in OTC, ASS1, and ASL with higher residual activity tend to result in milder phenotypes, while those causing near-complete loss are associated with early-onset, severe disease [6–8]. Irrespective of onset type, hyperammonemia can lead to irreversible brain injury and long-term neurocognitive sequelae [9–11]. Even in patients with only mild intellectual disability, challenges in executive functioning, emotional regulation, and social integration can significantly impair quality of life and autonomy.

Although the natural history and genetic spectrum of neonatal UCDs have been widely reported, little is known about the genotypes and long-term clinical trajectories of adults who have survived with UCDs into middle age. In this nationwide study, we investigated the genetic and clinical characteristics of adult long-term survivors with UCDs in Japan. We aimed to 1) identify their underlying pathogenic variants, 2) describe their clinical and intellectual outcomes, and 3) explore potential genotype–phenotype correlations that may inform long-term prognosis and patient support strategies.

## Materials and methods

### Study participants

To investigate the current clinical status of adult patients (aged ≥ 18 years) with urea cycle disorders (UCDs) in Japan, we conducted an initial nationwide, questionnaire-based survey in 2023. Questionnaires were distributed to 1,744 medical institutions, specifically targeting departments of general internal medicine, gastroenterology, neurology, general surgery, hepatobiliary and pancreatic surgery, and transplant surgery. The surveyed hospitals were primarily large-scale medical centers with more than 300 beds, functioning as regional healthcare hubs.

Clinicians were asked whether they had diagnosed or treated adult patients with UCDs, including carbamoyl phosphate synthetase 1 deficiency (CPS1D), N-acetylglutamate synthase deficiency (NAGSD), ornithine transcarbamylase deficiency (OTCD), argininosuccinate synthetase 1 deficiency (ASS1D), argininosuccinate lyase deficiency (ASLD), arginase 1 deficiency (ARG1D), or disorders involving ornithine transporter 1 (ORNT1) or citrin. Of the 1,744 institutions contacted, 774 (44%) responded. Among these, 42 institutions reported having cared for adult patients with UCDs. A follow-up questionnaire was subsequently sent to these 42 institutions, of which 22 (52%) provided additional responses.

As of 2024, we collected clinical data from 34 adult patients who had been diagnosed and/or treated for UCDs between January 2010 and December 2022. These included 3 patients with CPS1D, 20 with OTCD, 3 with ASS1D, 1 with ASLD, and 7 with hyperornithinemia-hyperammonemia-homocitrullinuria (HHH) syndrome. We then combined this dataset with data from 82 adult patients previously identified in a nationwide study conducted between January 2000 and March 2018 [9–12], resulting in a total of 116 adult UCD patients for analysis.

To avoid duplication, records from patients who had been seen at multiple institutions were consolidated, ensuring each patient was counted only once. The final cohort comprised 10 patients with CPS1D, 69 with OTCD, 17 with ASS1D, 9 with ASLD, 4 with ARG1D, and 7 with HHH syndrome. Diagnoses were established based on clinical history, family background, biochemical testing (plasma amino acids and urinary orotic acid), enzyme activity measurements, and/or molecular genetic analysis. Among the 116 patients, pathogenic variants were identified in 48 individuals: 2 with CPS1D, 34 with OTCD, 6 with ASS1D, 3 with ASLD, 1 with ARG1D, and 2 with HHH syndrome.

Cognitive function during long-term follow-up was assessed using standardized intelligence tests, including the Wechsler Adult Intelligence Scale, administered by licensed clinical psychologists or psychiatrists experienced in the assessment of neurocognitive disorders. An intelligence quotient (IQ) score of < 70 on these tests was classified as indicative of intellectual disability. In addition, neurodevelopmental disorders, including autism spectrum disorder, attention-deficit/hyperactivity disorder, and specific learning disorders, were diagnosed by qualified psychologists or psychiatrists based on comprehensive clinical evaluation and established diagnostic criteria.

### Statistical analysis

The chi-square test was used to evaluate the association between peak plasma ammonia levels (>360 μmol/L) and intellectual disability in patients with UCDs and in the OTCD subgroup (Supplementary Data 1).

### Ethics statement

This study was approved by the ethical committee of the Faculty of Life Science, Kumamoto University (Ethics. No. 1527). All procedures followed were in accordance with the ethical standards of the responsible committee on human experimentation (institutional and national) and with the Helsinki Declaration of 1975, as revised in 2000. Informed consent was obtained from all patients or their legal guardians for being included in the study.

## Results

### Genetic variants and clinical outcomes in UCDs

Table 1 summarizes the pathogenic variants, vital status, age at onset, peak plasma ammonia concentrations, and long-term clinical outcomes of 48 adult patients with UCDs, including 2 with CPS1D, 34 with OTCD, 6 with ASS1D, 3 with ASLD, 1 with ARG1D, and 2 with HHH syndrome. Although all patients survived into adulthood, three patients (CPS1-2, OTC-24, and OTC-33) died during the observation period. Their deaths were attributed to hyperammonemic encephalopathy resulting from the primary metabolic disorder. A diverse spectrum of pathogenic variants was identified, comprising nonsense, missense, frameshift, and splice-site mutations. Detailed annotations, including ClinVar and PolyPhen-2 predictions, ACMG classifications, and allele frequencies, are provided in Table 2 [13–25]. Further stratification of OTCD variants by variant type (e.g., missense vs. truncating variants) is provided in Supplementary Data 2.

**Table 1:** Pathogenic variants and long-term outcome of adult UCD patients in this study

Patient ID	Gender	Allele 1		Allele 2		Dead or Alive	Age at 2024.1	Onset time	Ammonia levels (μmol/L)	ID
		Nucleic acid	Amino acid	Nucleic acid	Amino acid					
CPS1-1	F	c.2397 G > A	p.M799I	c.2397 G > A	p.M799I	Alive	66y 9 m	Late (59 y 3 m)	318	-
CPS1-2	F	c.3422T > G	p.V1141G	c.3784C > T	p.R1262*	Dead (24y 8 m)	-	Late (14 y)	1,549.	**〇**
**OTC-1**	**M**	**c.77 G > A**	**p.Arg26Gln**			**Alive**	**21y 4 m**	**Neonatal (5 days)**	**2,349**	**〇**
**OTC-2**	**M**	**c.77 G > A**	**p.Arg26Gln**			**Alive**	**20y 4 m**	**Neonatal (1 day)**	**212**	-
OTC-3	F	c.119 G > A	p.Arg40His			Alive	60y 4 m	Late (52 y 1 m)	402	-
**OTC-4**	**M**	**c.119 G > A**	**p.Arg40His**			**Alive**	**41y**	**Late (35 y 2 m)**	**894**	**〇**
**OTC-5**	**M**	**c.119 G > A**	**p.Arg40His**			**Alive**	**51y 8 m**	**Late (44 y 5 m)**	**460**	-
**OTC-6**	**M**	**c.119 G > A**	**p.Arg40His**			**Alive**	**29y 4 m**	**Late (9 y)**	**640**	-
**OTC-7**	**M**	**c.119 G > A**	**p.Arg40His**			**Alive**	**19y 10 m**	**Late (3 y)**	**141**	-
**OTC-8**	**M**	**c.119 G > A**	**p.Arg40His**			**Alive**	**19y 8 m**	**Late (7 y)**	**445**	**〇**
OTC-9	F	c.140A > T	p.Asp47Ile			Alive	37y 4 m	Late (9 y)	90	-
**OTC-10**	**M**	**c.142T > G**	**p.Phe48Val**			**Alive**	**18y 6 m**	**Late (1 y 1 m)**	**404**	**〇**
**OTC-11**	**M**	**c.163T > G**	**p.Tyr55Asp**			**Alive**	**19y 1 m**	**Late (pre)**	**66**	-
**OTC-12**	**M**	**c.179C > T**	**p.Ser60Leu**			**Alive**	**18y 9 m**	**Late (8 m)**	**NA**	**〇**
OTC-13	F	c.254T > A	p.Ile85Asn			Alive	24y 9 m	Late (7 y 1 m)	119	-
**OTC-14**	**M**	**c.386 G > A**	**p.Arg129His**			**Alive**	**23y 9 m**	**Late (7 m)**	**274**	-
**OTC-15**	**M**	**c.515T > A**	**p.Ile172Asn**			**Alive**	**20 y**	**Late (11 m)**	**274**	**NA**
OTC-16	F	c.523_536del	p.Asp175Profs*5			Alive	27y 7 m	Late (5 y)	335	-
OTC-17	F	c.523_536del	p.Asp175Profs*5			Alive	31y 9 m	Late (8 m)	233	〇
OTC-18	F	c.533C > T	p.Thr178Met			Alive	39y 8 m	Late (32 y 2 m)	280	-
OTC-19	F	c.533C > T	p.Thr178Met			Alive	37y 6 m	Late (5 m)	116	-
OTC-20	F	c.542A > G	p.Glu181Gly			Alive	25y 4 m	Late (23 y 2 m)	424	〇
OTC-21	F	c.583 G > A	p.Gly195Arg			Alive	20y 3 m	Late (1 y 6 m)	1,174	〇
OTC-22	F	c.604C > T	p.His202Tyr			Alive	23y	Late (14 y)	344	-
OTC-23	F	c.617T > G	p.Met206Arg			Alive	19y 4 m	Late (10 m)	NA	NA
**OTC-24**	**M**	**c.622 G > A**	**p.Ala208Thr**			**Dead (46y 9 m)**	-	**Late (46 y 9 m)**	**483**	-
OTC-25	F	c.626C > T	p.Ala209Val			Alive	35y 8 m	Late (3 y)	117	-
OTC-26	F	c.643C > T	p.Leu215Phe			Alive	28y 10 m	Late (1 y)	180	-
**OTC-27**	**M**	**c.790A > G**	**p.Thr264Ala**			**Alive**	**40y 11 m**	**Late (4 y)**	**NA**	**〇**
**OTC-28**	**M**	**c.829C > T**	**p.Arg277Trp**			**Alive**	**39y 10 m**	**Late (10 y 1 m)**	**207**	-
**OTC-29**	**M**	**c.829C > T**	**p.Arg277Trp**			**Alive**	**33y 4 m**	**Late (2 y)**	**183**	-
OTC-30	F	c.894 G > A	p.Trp298*			Alive	26y 3 m	Late (11 m)	570	-
**OTC-31**	**M**	**c.912 G > C**	**p.Leu304Phe**			**Alive**	**34y 5 m**	**Late (1 y 3 m)**	**169**	-
OTC-32	F	c.940 G > T	p.Glu314*			Alive	19y 3 m	Late (2 y 2 m)	166	〇
OTC-33	F	c.959 G > T	p.Arg320Leu			Dead (61y 10 m)	-	Late (61 y 10 m)	1,115	-
OTC-34	F	Ex1-6dup	-			Alive	27y 10 m	Late (4 y)	129	-
ASS-1	F	c.421-2A > G	-	c.421-2A > G		Alive	40y 6 m	Neonatal (3 days)	404	〇
ASS-2	F	c.421-2A > G	-	c.421-2A > G	-	Alive	35y 9 m	Neonatal (1 day)	336	-
ASS-3	F	c.421-2A > G	-	c.814C > T	p.Arg272Cys	Alive	18y 2 m	Neonatal (3 days)	1,033	〇
**ASS-4**	**M**	**c.421-2A > G**	-	**c.910C > T**	**p.Arg304Trp**	**Alive**	**18y 6 m**	**Neonatal (6 days)**	**834**	-
ASS-5	F	c.421-2A > G	-	c.1063A > G	p.Lys355Glu	Alive	24y 7 m	Late (13y 7 m)	306	〇
**ASS-6**	**M**	**c.794 G > A**	**p.Arg265His**	**ND**		**Alive**	**21y 5 m**	**Late (1 y 6 m)**	**129**	**〇**
**ASL-1**	**M**	**c.91 G > A**	**p.Asp31Asn**	**c.918+5 G > A**	-	**Alive**	**29y 2 m**	**Late (6 m)**	**135**	**〇**
**ASL-2**	**M**	**c.91 G > A**	**p.Asp31Asn**	**c.1251-1 G > C**	-	**Alive**	**21y 10 m**	**Late (14 y 1 m)**	**499**	**〇**
**ASL-3**	**M**	**c. 347A > G**	**p.Gln116Arg**	**c.637C > T**	p.Arg213*	**Alive**	**35y 6 m**	**Neonatal (8 days)**	**312**	**〇**
ARG1-1	F	c.122-125insCTTA	p.Lys41Thrfs*2	c.298 G > A	p.Asp100Asn	Alive	20y 1 m	Late (10 y 9 m)	73	〇
**HHH-1**	**M**	**c.535C > T**	p.Arg179*	**c.535C > T**	p.Arg179*	**Alive**	**52y 11 m**	**Late (primary school)**	**525**	**〇**
HHH-2	F	c.823C > T	p.Arg275*	c.823C > T	p.Arg275*	Alive	**52y 5 m**	Late (5 m)	108	〇

NA: Not available, ID: Intellectual disability, pre: prenatal diagnosis, M: Male (Bold), F: Female. Late (pre): This variant is generally considered to be associated with late-onset OTCD, however, the patient carrying this variant was diagnosed prenatally.” The ages of living OTCD patients as of January 2024 were a median of 22 years and 7 months (range: 18 years 6 months to 51 years 8 months) in males (*N* = 16) and 27 years and 8 months (range: 19 years 3 months to 60 years 4 months) in females (*N* = 16), respectively. The ages of living ASSD patients were a median of 23 years (range: 18 years 2 months to 40 years 6 months)

**Table 2:** The variants detected in adult patients with UCDs in Japan

Variant No	Nucleic acid	Amino acid	ClinVar	Polyphen-2 (Score)	ACMG interpretation		Allele frequency		References
					Category	Classification	In the patient	jMorp	
CPS-1	c.2397 G > A	p.Met799Ile	NR	Probably damaging (0.998)	PM2, PP3	Uncertain significance	0.50 (2/4)	NR	14
CPS-2	c.3422T > G	p.Val1141Gly	NR	Probably damaging (1.000)	PM2, PP3	Uncertain significance	0.25 (1/4)	NR	14
CPS-3	c.3784C > T	p.Arg1262*	P	NA	PVS1, PM2	Likely pathogenic	0.25 (1/4)	0.000133	14
OTC-1	c.77 G > A	p.Arg26Gln	P/LP	Benign (0.002)	PS3, PM2, PM5, PP3, BP4	Likely pathogenic	0.0392 (2/51)	NR	14
OTC-2	c.119 G > A	p.Arg40His	P/LP	Possibly damaging (0.668)	PS3, PM2, PM5, PP1	Likely pathogenic	0.0196 (1/51)	NR	14, 19
OTC-3	c.140A > T	p.Asn47Ile	P	Possibly damaging (0.477)	PM2	Uncertain significance	0.0196 (1/51)	NR	14
OTC-4	c.142T > G	p.Phe48Val	NR	Possibly damaging (0.883)	PM2	Uncertain significance	0.0196 (1/51)	NR	14
OTC-5	c.163T > G	p.Tyr55Asp	P	Probably damaging (0.995)	PM2, PP3	Uncertain significance	0.0196 (1/51)	NR	21
OTC-6	c.179C > T	p.Ser60Leu	P	Possibly damaging (0.916)	PM2	Uncertain significance	0.0196 (1/51)	NR	14
OTC-7	c.254T > A	p.Ile85Asn	NR	Probably damaging (1.000)	PM2, PP3	Uncertain significance	0.0196 (1/51)	NR	14
OTC-8	c.386 G > A	p.Arg129His	P	Probably damaging (1.000)	PS3, PM2, PM5, PP3, PP4	Pathogenic	0.0196 (1/51)	NR	17, 24
OTC-9	c.515T > A	p.Ile172Asn	NR	Probably damaging (1.000)	PM2, PP3	Uncertain significance	0.0196 (1/51)	NR	14
OTC-10	c.523_536del	p.Asp175Profs*5	NR	NA	PVS1, PM2	Likely pathogenic	0.0392 (2/51)	NR	14
OTC-11	c.533C > T	p.Thr178Met	P	Probably damaging (1.000)	PM2, PP3	Uncertain significance	0.0392 (2/51)	NR	14
OTC-12	c.542A > G	p.Glu181Gly	P	Probably damaging (0.969)	PM2, PP3	Uncertain significance	0.0196 (1/51)	NR	22
OTC-13	c.583 G > A	p.Gly195Arg	P	Probably damaging (1.000)	PM2, PP3	Uncertain significance	0.0196 (1/51)	NR	14
OTC-14	c.604C > T	p.His202Tyr	P	Probably damaging (0.998)	PM2, PP3	Uncertain significance	0.0196 (1/51)	NR	14
OTC-15	c.617T > G	p.Met206Arg	P	Possibly damaging (0.722)	PM2	Uncertain significance	0.0196 (1/51)	NR	14
OTC-16	c.622 G > A	p.Ala208Thr	P	Probably damaging (0.999)	PM2, PP3	Uncertain significance	0.0196 (1/51)	NR	23
OTC-17	c.626C > T	p.Ala209Val	P	Probably damaging (1.000)	PM2, PP3	Uncertain significance	0.0196 (1/51)	NR	14
OTC-18	c.643C > T	p.Leu215Phe	P	Probably damaging (0.968)	PM2, PP3	Uncertain significance	0.0196 (1/51)	NR	14
OTC-19	c.790A > G	p.Thr264Ala	P	Probably damaging (0.998)	PM2, PP3	Uncertain significance	0.0196 (1/51)	NR	14
OTC-20	c.829C > T	p.Arg277Trp	P	Probably damaging (1.000)	PM2, PP3	Uncertain significance	0.0392 (2/51)	NR	14
OTC-21	c.894 G > A	p.Trp298*	NR	NA	PVS1, PM2	Likely pathogenic	0.0196 (1/51)	NR	14
OTC-22	c.912 G > C	p.Leu304Phe	NR	Probably damaging (0.996)	PM2, PP3	Uncertain significance	0.0196 (1/51)	NR	14
OTC-23	c.940 G > T	p.Glu314*	NR	NA	PVS1, PM2	Likely pathogenic	0.0196 (1/51)	NR	14
OTC-24	c.959 G > T	p.Arg320Leu	P	Probably damaging (0.999)	PM2, PM5, PP3	Uncertain significance	0.0196 (1/51)	NR	13, 25
OTC-25	Ex1-6dup	-	NR	NA	PM2	Uncertain significance	0.0196 (1/51)	NR	18
ASS-1	c.421-2A > G	-	P	NA	PVS1, PS4, PM2	Pathogenic	0.5833 (7/12)	0.000667	14
ASS-2	c.794 G > A	p.Arg265His	P/LP	Possibly damaging (0.915)	PM2	Uncertain significance	0.0833 (1/12)	NR	14
ASS-3	c.814C > T	p.Arg272Cys	P	Probably damaging (0.991)	PM2, PP3	Uncertain significance	0.0833 (1/12)	0.000058	14
ASS-4	c.910C > T	p.Arg304Trp	P	Possibly damaging (0.868)	PM2	Uncertain significance	0.0833 (1/12)	0.000501	14
ASS-5	c.1063A > G	p.Lys355Glu	NR	Benign (0.321)	PM2, BP4	Uncertain significance	0.0833 (1/12)	0.000017	14
ASL-1	c.91 G > A	p.Asp31Asn	P/LP	Probably damaging (0.997)	PM2, PP3	Uncertain significance	0.3333 (2/6)	0.000017	14
ASL-2	c.347A > G	p.Gln116Arg	NR	Probably damaging (1.000)	PM2, PP3	Uncertain significance	0.1666 (1/6)	0.000033	14
ASL-3	c.637C > T	p.Arg213*	P	NA	PVS1, PM2	Likely pathogenic	0.1666 (1/6)	NR	14
ASL-4	c.918+5 G > A	-	CP	NA	PM2	Uncertain significance	0.1666 (1/6)	0.000008	14
ASL-5	c.1251-1 G > C	-	NR	NA	PVS1, PM2	Likely pathogenic	0.1666 (1/6)	NR	14
ARG-1	c.122-125insCTTA	p.Lys41Thrfs*2	NR	NA	PVS1, PM2	Likely pathogenic	0.5000 (1/2)	NR	15
ARG-2	c.298 G > A	p.Asp100Asn	NR	Probably damaging (0.999)	PM2, PP3	Uncertain significance	0.5000 (1/2)	NR	14
HHH-1	c.535C > T	p.Arg179*	P	NA	PVS1, PM2	Likely pathogenic	0.5000 (2/4)	0.000309	20
HHH-2	c.823C > T	p.Arg275*	P/LP	NA	PVS1, PM2	Likely pathogenic	0.5000 (2/4)	0.000025	18

NA: Not available, NR: not registered, P: Pathogenic, LP: Likely pathogenic, CP: Conflicting classifications of pathogenicity. jMorp: Japanese Multi-Omics Reference Panel

The most frequent recurrent variant was c.119 G > A (p.Arg40His) in the *OTC* gene, found in six unrelated male patients. Peak plasma ammonia levels during the disease course ranged widely from 66 to 2,349 μmol/L. Intellectual disability (ID) was present in 19 of 44 evaluable patients (43.2%) and was significantly associated with peak ammonia concentrations exceeding 360 μmol/L (*p* = 0.046). This association was particularly strong among patients with OTCD (*N* = 30, *p* = 0.018; see Supplementary Data 1). Patients with the *OTC* c.119 G > A (p.Arg40His) variant exhibited high ammonia levels, with a median of 423 μmol/L (interquartile range: 237–456 μmol/L).

Both patients with HHH syndrome (HHH-1 and HHH-2) shared homozygous nonsense variants in the *SLC25A15* gene (c.535C > T or c.823C > T) and were diagnosed during early childhood. Notably, patients with ASLD or HHH syndrome frequently developed intellectual disability regardless of the age at disease onset or the severity of hyperammonemia.

### Intellectual disability and functional outcomes

Table 3, 4, 5 and 6 outlines the clinical features associated with each genetic variant. These included neurological, gastrointestinal, hematological, hepatic, and developmental or neuropsychiatric manifestations. Neurological involvement—such as hyperammonemic coma, seizures, and abnormal findings on electroencephalogram or neuroimaging—was observed across multiple genotypes. Gastrointestinal symptoms included hepatomegaly, protein intolerance, anorexia, vomiting, and liver disorder, often evidenced by elevated transaminases or imaging abnormalities. Liver transplantation was required in some patients with severe phenotypes, particularly those with CPS1D or OTCD.

**Table 3:** The variants and clinical manifestations in adult UCD patients. Neurological manifestations

Patient ID	Gender	Allele 1		Allele 2		Hypo-glycemia	Hyperammonemic coma	Seizure	Hyper- or Hypotonia	Abnormal brain MRI or CT	Abnormal EEG	CP like symptoms
		Nucleic acid	Amino acid	Nucleic acid	Amino acid							
CPS1-1	F	c.2397 G > A	p.Met799Ile	c.2397 G > A	p.Met799Ile	-	〇	-	-	〇	-	-
CPS1-2	F	c.3422T > G	p.Val1141Gly	c.3784C > T	p.Arg1262*	-	〇	〇	〇	〇	〇	〇
**OTC-1**	**M**	**c.77 G > A**	**p.Arg26Gln**			-	**〇**	**〇**	**〇**	**〇**	**〇**	**〇**
**OTC-2**	**M**	**c.77 G > A**	**p.Arg26Gln**			-	-	-	-	**NA**	**NA**	-
OTC-3	F	c.119 G > A	p.Arg40His			-	-	-	-	-	-	-
**OTC-4**	**M**	**c.119 G > A**	**p.Arg40His**			**〇**	**〇**	**〇**	**〇**	**〇**	**〇**	-
**OTC-5**	**M**	**c.119 G > A**	**p.Arg40His**			-	-	-	-	-	**NA**	-
**OTC-6**	**M**	**c.119 G > A**	**p.Arg40His**			-	**〇**	-	-	-	-	-
**OTC-7**	**M**	**c.119 G > A**	**p.Arg40His**			-	**〇**	**〇**	-	-	**〇**	-
**OTC-8**	**M**	**c.119 G > A**	**p.Arg40His**			-	**〇**	**〇**	-	-	-	-
OTC-9	F	c.140A > T	p.Asp47Ile			-	〇	-	-	-	-	-
**OTC-10**	**M**	**c.142T > G**	**p.Phe48Val**			-	**NA**	**NA**	**NA**	**NA**	**NA**	**NA**
**OTC-11**	**M**	**c.163T > G**	**p.Tyr55Asp**			-	-	-	-	-	-	-
**OTC-12**	**M**	**c.179C > T**	**p.Ser60Leu**			-	**〇**	-	-	**〇**	-	-
OTC-13	F	c.254T > A	p.Ile85Asn			-	〇	-	-	-	NA	-
**OTC-14**	**M**	**c.386 G > A**	**p.Arg129His**			-	**〇**	**〇**	-	**NA**	**NA**	-
**OTC-15**	**M**	**c.515T > A**	**p.Ile172Asn**			-	**〇**	-	-	-	-	-
OTC-16	F	c.523_536del	p.Asp175Profs*5			-	-	-	-	NA	NA	-
OTC-17	F	c.523_536del	p.Asp175Profs*5			-	-	-	-	NA	NA	-
OTC-18	F	c.533C > T	p.Thr178Met			-	〇	-	-	〇	〇	-
OTC-19	F	c.533C > T	p.Thr178Met			-	〇	-	-	-	-	-
OTC-20	F	c.542A > G	p.Glu181Gly			-	〇	-	-	-	-	-
OTC-21	F	c.583 G > A	p.Gly195Arg			-	〇	〇	〇	〇	〇	〇
OTC-22	F	c.604C > T	p.His202Tyr			-	〇	-	-	NA	NA	-
OTC-23	F	c.617T > G	p.Met206Arg			-	NA	NA	NA	NA	NA	NA
**OTC-24**	**M**	**c.622 G > A**	**p.Ala208Thr**			-	**〇**	-	-	-	-	-
OTC-25	F	c.626C > T	p.Ala209Val			-	-	-	-	-	NA	-
OTC-26	F	c.643C > T	p.Leu215Phe			-	〇	-	-	-	-	-
**OTC-27**	**M**	**c.790A > G**	**p.Thr264Ala**			-	**〇**	-	-	-	-	-
**OTC-28**	**M**	**c.829C > T**	**p.Arg277Trp**			-	**〇**	-	-	-	**NA**	-
**OTC-29**	**M**	**c.829C > T**	**p.Arg277Trp**			-	-	-	-	**NA**	**NA**	-
OTC-30	F	c.894 G > A	p.Trp298*			-	-	-	-	NA	NA	-
**OTC-31**	**M**	**c.912 G > C**	**p.Leu304Phe**			-	-	-	-	-	-	-
OTC-32	F	c.940 G > T	p.Glu314*			-	〇	-	-	-	-	-
OTC-33	F	c.959 G > T	p.Arg320Leu			-	〇	〇	〇	〇	〇	-
OTC-34	F	Ex1-6dup	-			-	-	-	-	NA	NA	-
ASS-1	F	c.421-2A > G	-	c.421-2A > G		-	〇	-	-	〇	-	-
ASS-2	F	c.421-2A > G	-	c.421-2A > G	-	-	〇	-	-	〇	-	-
ASS-3	F	c.421-2A > G	-	c.814C > T	p.Arg272Cys	-	〇	〇	-	〇	〇	-
**ASS-4**	**M**	**c.421-2A > G**	-	**c.910C > T**	**p.Arg304Trp**	-	**〇**	-	-	**NA**	**NA**	-
ASS-5	F	c.421-2A > G	-	c.1063A > G	p.Lys355Glu	-	〇	-	〇	-	〇	-
**ASS-6**	**M**	**c.794 G > A**	**p.Arg265His**	**ND**		-	**〇**	-	-	-	-	-
**ASL-1**	**M**	**c.91 G > A**	**p.Asp31Asn**	**c.918+5 G > A**	-	-	**〇**	**〇**	-	-	**NA**	-
**ASL-2**	**M**	**c.91 G > A**	**p.Asp31Asn**	**c.1251-1 G > C**	-	-	**〇**	**〇**	-	**〇**	**〇**	-
**ASL-3**	**M**	**c. 347A > G**	**p.Gln116Arg**	**c.637C > T**	p.Arg213*	-	**〇**	**〇**	**〇**	**〇**		-
ARG1-1	F	c.122-125insCTTA	p.Lys41Thrfs*2	c.298 G > A	p.Asp100Asn	-	〇	〇	-	NA	NA	-
**HHH-1**	**M**	**c.535C > T**	p.Arg179*	**c.535C > T**	p.Arg179*	-	**〇**	**〇**	-	**〇**	**NA**	-
HHH-2	F	c.823C > T	p.Arg275*	c.823C > T	p.Arg275*	-	NA	〇	NA	〇	〇	NA

NA: Not available, ID: Intellectual disability, CP: cerebral palsy, EEG: electroencephalogram. 〇: Yes, -: No, Bold: Male. Cerebral palsy like symptoms encompass motor impairments, including spasticity and abnormal gait abnormalities

**Table 4:** B gastrointestinal manifestations

Patient ID	Allele 1		Allele 2		Hepatomegaly	Spleen enlargement	Aversion of protein	Anorexia	Nausea or Vomiting	Intractable diarrhea	Elevated transaminase	Liver damage (abnormal imaging test results)	LT
	Nucleic acid	Amino acid	Nucleic acid	Amino acid									
CPS1-1	c.2397 G > A	p.Met799Ile	c.2397 G > A	p.Met799Ile	-	-	-	-	-	-	-	-	〇
CPS1-2	c.3422T > G	p.Val1141Gly	c.3784C > T	p.Arg1262*	-	-	NA	-	〇	-	〇	NA	-
**OTC-1**	**c.77 G > A**	**p.Arg26Gln**			**〇**	-	**NA**	-	-	-	**〇**	-	-
**OTC-2**	**c.77 G > A**	**p.Arg26Gln**			-	-	**NA**						**〇**
OTC-3	c.119 G > A	p.Arg40His			-	-	-						-
**OTC-4**	**c.119 G > A**	**p.Arg40His**			-	-	**〇**						-
**OTC-5**	**c.119 G > A**	**p.Arg40His**			-	-	-						-
**OTC-6**	**c.119 G > A**	**p.Arg40His**			-	-	-						-
**OTC-7**	**c.119 G > A**	**p.Arg40His**			-	-	**〇**						-
**OTC-8**	**c.119 G > A**	**p.Arg40His**			-	-	**NA**						-
OTC-9	c.140A > T	p.Asp47Ile			-	-	〇	〇	〇	NA	〇	NA	〇
**OTC-10**	**c.142T > G**	**p.Phe48Val**			-	-	**NA**	**NA**	-	-	**〇**	**NA**	**〇**
**OTC-11**	**c.163T > G**	**p.Tyr55Asp**			-	-	-	-	**〇**	-	**〇**	**NA**	-
**OTC-12**	**c.179C > T**	**p.Ser60Leu**			-	-	-	-	-	-	-	-	**〇**
OTC-13	c.254T > A	p.Ile85Asn			-	-	-	-	〇	-	〇	-	-
**OTC-14**	**c.386 G > A**	**p.Arg129His**			**〇**	-	-	**〇**	**〇**	-	-	-	**〇**
**OTC-15**	**c.515T > A**	**p.Ile172Asn**			-	-	-	**〇**	**〇**	**〇**	**〇**	**〇**	-
OTC-16	c.523_536del	p.Asp175Profs*5			-	-	-	-	-	-	-	-	-
OTC-17	c.523_536del	p.Asp175Profs*5			-	-	-	-	-	-	-	-	-
OTC-18	c.533C > T	p.Thr178Met			-	-	〇	-	〇	-	-	-	-
OTC-19	c.533C > T	p.Thr178Met			-	-	〇	-	-	〇	〇	-	-
OTC-20	c.542A > G	p.Glu181Gly			-	-	〇	-	-	-	-	-	〇
OTC-21	c.583 G > A	p.Gly195Arg			-	-	NA	NA	-	-	〇	-	-
OTC-22	c.604C > T	p.His202Tyr			-	-	-	-	-	-	-	-	-
OTC-23	c.617T > G	p.Met206Arg			-	-	NA	NA	NA	NA	NA	NA	-
**OTC-24**	**c.622 G > A**	**p.Ala208Thr**			-	-	-	-	-	-	-	-	-
OTC-25	c.626C > T	p.Ala209Val			-	-	〇	-	〇	-	〇	-	-
OTC-26	c.643C > T	p.Leu215Phe			-	-	-	-	〇	-	〇	-	-
**OTC-27**	**c.790A > G**	**p.Thr264Ala**			-	-	-	-	**〇**	-	-	-	-
**OTC-28**	**c.829C > T**	**p.Arg277Trp**			-	-	-	-	**〇**	-	-	-	-
**OTC-29**	**c.829C > T**	**p.Arg277Trp**			-	-	**NA**	-	**〇**	-	**〇**	-	-
OTC-30	c.894 G > A	p.Trp298*			-	-	〇	-	-	-	-	-	-
**OTC-31**	**c.912 G > C**	**p.Leu304Phe**			-	-	**NA**	**〇**	**〇**	-	-	-	**〇**
OTC-32	c.940 G > T	p.Glu314*			-	-	〇	〇	〇	-	〇	-	〇
OTC-33	c.959 G > T	p.Arg320Leu			-	-	-	〇	〇	-	-	-	-
OTC-34	Ex1-6dup	-			-	-	-	-	-	-	-	-	-
ASS-1	c.421-2A > G	-	c.421-2A > G		-	-	NA	-	〇	-	〇	〇	-
ASS-2	c.421-2A > G	-	c.421-2A > G	-	-	-	NA	-	〇	-	〇	〇	-
ASS-3	c.421-2A > G	-	c.814C > T	p.Arg272Cys	-	-	NA	-	-	-	〇	NA	-
**ASS-4**	**c.421-2A > G**	-	**c.910C > T**	**p.Arg304Trp**	-	-	**NA**	-	-	-	-	**NA**	-
ASS-5	c.421-2A > G	-	c.1063A > G	p.Lys355Glu	-	-	-	-	-	-	-	NA	-
**ASS-6**	**c.794 G > A**	**p.Arg265His**	**ND**		-	-	-	-	-	-	-	-	-
**ASL-1**	**c.91 G > A**	**p.Asp31Asn**	**c.918+5 G > A**	-	**〇**	-	**NA**	**NA**	**NA**	**NA**	-	**NA**	-
**ASL-2**	**c.91 G > A**	**p.Asp31Asn**	**c.1251-1 G > C**	-	-	-	**〇**	-	**〇**	**〇**	**〇**	-	-
**ASL-3**	**c. 347A > G**	**p.Gln116Arg**	**c.637C > T**	p.Arg213*	**〇**	-	**〇**	**〇**	**〇**	-	**〇**	**〇**	-
ARG1-1	c.122-125insCTTA	p.Lys41Thrfs*2	c.298 G > A	p.Asp100Asn	-	-	〇	-	-	-	〇	NA	-
**HHH-1**	**c.535C > T**	p.Arg179*	**c.535C > T**	p.Arg179*	-	-	-	-	-	-	-	-	-
HHH-2	c.823C > T	p.Arg275*	c.823C > T	p.Arg275*	NA	NA	NA	NA	NA	NA	〇	NA	-

NA: Not available, LT: Liver transplant

**Table 5:** C hematological manifestations

Patient ID	Allele 1		Allele 2		Leukopenia	Anemia	Thrombocytopenia	Bleeding tendency
	Nucleic acid	Amino acid	Nucleic acid	Amino acid				
CPS1-1	c.2397 G > A	p.Met799Ile	c.2397 G > A	p.Met799Ile	〇	〇	〇	-
CPS1-2	c.3422T > G	p.Val1141Gly	c.3784C > T	p.Arg1262*	-	-	-	-
**OTC-1**	**c.77 G > A**	**p.Arg26Gln**			-	-	-	-
**OTC-2**	**c.77 G > A**	**p.Arg26Gln**			**〇**	**NA**	**NA**	**NA**
OTC-3	c.119 G > A	p.Arg40His			-	-	-	-
**OTC-4**	**c.119 G > A**	**p.Arg40His**			-	-	-	-
**OTC-5**	**c.119 G > A**	**p.Arg40His**			-	-	-	-
**OTC-6**	**c.119 G > A**	**p.Arg40His**			-	-	-	-
**OTC-7**	**c.119 G > A**	**p.Arg40His**			**NA**	**NA**	**NA**	**NA**
**OTC-8**	**c.119 G > A**	**p.Arg40His**			-	-	-	-
OTC-9	c.140A > T	p.Asp47Ile			-	-	-	-
**OTC-10**	**c.142T > G**	**p.Phe48Val**			-	-	**〇**	-
**OTC-11**	**c.163T > G**	**p.Tyr55Asp**			**NA**	**NA**	**NA**	**NA**
**OTC-12**	**c.179C > T**	**p.Ser60Leu**			-	-	-	-
OTC-13	c.254T > A	p.Ile85Asn			-	-	-	-
**OTC-14**	**c.386 G > A**	**p.Arg129His**			-	-	-	-
**OTC-15**	**c.515T > A**	**p.Ile172Asn**			-	-	-	-
OTC-16	c.523_536del	p.Asp175Profs*5			-	-	-	-
OTC-17	c.523_536del	p.Asp175Profs*5			-	-	-	-
OTC-18	c.533C > T	p.Thr178Met			-	-	-	-
OTC-19	c.533C > T	p.Thr178Met			-	-	NA	-
OTC-20	c.542A > G	p.Glu181Gly			-	-	-	-
OTC-21	c.583 G > A	p.Gly195Arg			-	-	-	-
OTC-22	c.604C > T	p.His202Tyr			-	-	-	-
OTC-23	c.617T > G	p.Met206Arg			NA	NA	NA	NA
**OTC-24**	**c.622 G > A**	**p.Ala208Thr**			-	-	-	-
OTC-25	c.626C > T	p.Ala209Val			-	-	-	-
OTC-26	c.643C > T	p.Leu215Phe			-	-	-	〇
**OTC-27**	**c.790A > G**	**p.Thr264Ala**			-	-	-	-
**OTC-28**	**c.829C > T**	**p.Arg277Trp**			**NA**	**NA**	**NA**	**NA**
**OTC-29**	**c.829C > T**	**p.Arg277Trp**			-	**〇**	-	-
OTC-30	c.894 G > A	p.Trp298*			-	-	-	-
**OTC-31**	**c.912 G > C**	**p.Leu304Phe**			-	-	-	-
OTC-32	c.940 G > T	p.Glu314*			-	-	-	-
OTC-33	c.959 G > T	p.Arg320Leu			-	-	-	-
OTC-34	Ex1-6dup	-			-	-	-	-
ASS-1	c.421-2A > G	-	c.421-2A > G		-	-	-	-
ASS-2	c.421-2A > G	-	c.421-2A > G	-	-	-	-	-
ASS-3	c.421-2A > G	-	c.814C > T	p.Arg272Cys	-	-	-	〇
**ASS-4**	**c.421-2A > G**	-	**c.910C > T**	**p.Arg304Trp**	-	**〇**	-	-
ASS-5	c.421-2A > G	-	c.1063A > G	p.Lys355Glu	-	-	-	-
**ASS-6**	**c.794 G > A**	**p.Arg265His**	**ND**		-	-	-	-
**ASL-1**	**c.91 G > A**	**p.Asp31Asn**	**c.918+5 G > A**	-	-	-	-	-
**ASL-2**	**c.91 G > A**	**p.Asp31Asn**	**c.1251-1 G > C**	-	**〇**	**〇**	-	-
**ASL-3**	**c. 347A > G**	**p.Gln116Arg**	**c.637C > T**	p.Arg213*	**〇**	**〇**	**〇**	-
ARG1-1	c.122-125insCTTA	p.Lys41Thrfs*2	c.298 G > A	p.Asp100Asn	-	-	-	-
**HHH-1**	**c.535C > T**	p.Arg179*	**c.535C > T**	p.Arg179*	-	-	-	-
HHH-2	c.823C > T	p.Arg275*	c.823C > T	p.Arg275*	-	-	-	-

NA: Not available, ID: Intellectual disability

**Table 6:** D developmental disorders

Patient ID	Allele 1		Allele 2		Autism	ADHD	Learning disorder	Adjustment disorder	Communication disorder	Mood disorder	ID	Final academic background
	Nucleic acid	Amino acid	Nucleic acid	Amino acid								
CPS1-1	c.2397 G > A	p.Met799Ile	c.2397 G > A	p.Met799Ile	-	-	-	-	-	-	-	High school
CPS1-2	c.3422T > G	p.Val1141Gly	c.3784C > T	p.Arg1262*	〇	-	-	-	〇	-	〇	High school
**OTC-1**	**c.77 G > A**	**p.Arg26Gln**			**NA**	**NA**	**NA**	**NA**	**〇**	**NA**	**〇**	**Special support school**
**OTC-2**	**c.77 G > A**	**p.Arg26Gln**			-	-	-	-	-	-	-	**High school**
OTC-3	c.119 G > A	p.Arg40His			-	-	-	-	-	-	-	NA
**OTC-4**	**c.119 G > A**	**p.Arg40His**			-	-	-	**〇**	-	**〇**	**〇**	**Bachelor course**
**OTC-5**	**c.119 G > A**	**p.Arg40His**			-	-	-	-	-	-	-	**Professional training college**
**OTC-6**	**c.119 G > A**	**p.Arg40His**			-	-	-	-	-	-	-	**Master course**
**OTC-7**	**c.119 G > A**	**p.Arg40His**			-	-	-	-	-	-	-	**High school**
**OTC-8**	**c.119 G > A**	**p.Arg40His**			-	-	-	-	**〇**	-	**〇**	**Junior high school**
OTC-9	c.140A > T	p.Asp47Ile			-	-	-	-	-	-	-	Bachelor course
**OTC-10**	**c.142T > G**	**p.Phe48Val**			**NA**	**NA**	**NA**	**NA**	**〇**	**NA**	**〇**	**Special support school**
**OTC-11**	**c.163T > G**	**p.Tyr55Asp**			-	-	-	-	-	-	-	**Junior high school**
**OTC-12**	**c.179C > T**	**p.Ser60Leu**			-	-	-	-	**〇**	-	**〇**	**Special support school**
OTC-13	c.254T > A	p.Ile85Asn			NA	NA	NA	NA	-	NA	-	Special support school
**OTC-14**	**c.386 G > A**	**p.Arg129His**			-	-	-	-	-	-	-	**Master course**
**OTC-15**	**c.515T > A**	**p.Ile172Asn**			-	-	-	-	**NA**	-	**NA**	**Junior high school**
OTC-16	c.523_536del	p.Asp175Profs*5			-	-	-	-	-	-	-	Professional training college
OTC-17	c.523_536del	p.Asp175Profs*5			-	-	-	-	〇	-	〇	Professional training college
OTC-18	c.533C > T	p.Thr178Met			-	-	-	-	-	-	-	High school
OTC-19	c.533C > T	p.Thr178Met			-	-	-	-	-	-	-	High school
OTC-20	c.542A > G	p.Glu181Gly			NA	NA	〇	NA	〇	NA	〇	Professional training college
OTC-21	c.583 G > A	p.Gly195Arg			NA	NA	NA	NA	〇	NA	〇	Special support school
OTC-22	c.604C > T	p.His202Tyr			-	-	-	NA	-	-	-	Correspondence high school
OTC-23	c.617T > G	p.Met206Arg			NA	NA	NA	NA	NA	NA	NA	Junior high school
**OTC-24**	**c.622 G > A**	**p.Ala208Thr**			**NA**	**NA**	**NA**	**NA**	-	**NA**	-	**High school**
OTC-25	c.626C > T	p.Ala209Val			-	-	-	-	-	-	-	High school
OTC-26	c.643C > T	p.Leu215Phe			-	-	-	-	-	-	-	Junior college
**OTC-27**	**c.790A > G**	**p.Thr264Ala**			-	-	-	-	**〇**	-	**〇**	**NA**
**OTC-28**	**c.829C > T**	**p.Arg277Trp**			-	-	-	-	-	-	-	**Bachelor course**
**OTC-29**	**c.829C > T**	**p.Arg277Trp**			-	-	-	-	-	-	-	**NA**
OTC-30	c.894 G > A	p.Trp298*			-	-	-	-	-	-	-	Professional training college
**OTC-31**	**c.912 G > C**	**p.Leu304Phe**			-	-	-	**〇**	-	-	-	**Bachelor course**
OTC-32	c.940 G > T	p.Glu314*			-	-	-	-	〇	-	〇	Bachelor course
OTC-33	c.959 G > T	p.Arg320Leu			-	-	-	-	-	-	-	NA
OTC-34	Ex1-6dup	-			-	-	-	-	-	-	-	NA
ASS-1	c.421-2A > G	-	c.421-2A > G		-	-	-	-	〇	-	〇	Special support school
ASS-2	c.421-2A > G	-	c.421-2A > G	-	-	-	-	-	-	-	-	Professional training college
ASS-3	c.421-2A > G	-	c.814C > T	p.Arg272Cys	-	〇	NA	NA	〇	-	〇	Junior high school
**ASS-4**	**c.421-2A > G**	-	**c.910C > T**	**p.Arg304Trp**	-	-	-	-	-	-	-	**Junior high school**
ASS-5	c.421-2A > G	-	c.1063A > G	p.Lys355Glu	-	-	-	-	〇	-	〇	Special support school
**ASS-6**	**c.794 G > A**	**p.Arg265His**	**ND**		-	-	**〇**	-	**〇**	-	**〇**	**Special support school**
**ASL-1**	**c.91 G > A**	**p.Asp31Asn**	**c.918+5 G > A**	-	-	-	-	-	**〇**	-	**〇**	**Special support school**
**ASL-2**	**c.91 G > A**	**p.Asp31Asn**	**c.1251-1 G > C**	-	-	-	**〇**	-	**〇**	-	**〇**	**Special support school**
**ASL-3**	**c. 347A > G**	**p.Gln116Arg**	**c.637C > T**	p.Arg213*	**NA**	**NA**	**NA**	**NA**	**〇**	**NA**	**〇**	**Special support school**
ARG1-1	c.122-125insCTTA	p.Lys41Thrfs*2	c.298 G > A	p.Asp100Asn	-	-	-	-	〇	-	〇	Junior high school
**HHH-1**	**c.535C > T**	p.Arg179*	**c.535C > T**	p.Arg179*	-	-	-	-	**〇**	-	**〇**	**Correspondence high school**
HHH-2	c.823C > T	p.Arg275*	c.823C > T	p.Arg275*	NA	NA	NA	NA	〇	NA	〇	High school

ADHD < b urid=“c3cef16850e7”>:  Attention-deficit/hyperactivity disorder, NA: not available

Among the 21 patients with intellectual disability, 95% (20/21) had coexisting communication impairments. In contrast, 86% (18/21) of patients without intellectual disability were able to graduate from a regular high school. Of note, patient OTC-4 developed mild intellectual disability after experiencing a hyperammonemic crisis following achievement of Bachelor’s degree.

### Genotype–phenotype correlation in male OTCD patients

**Fig. 1:**
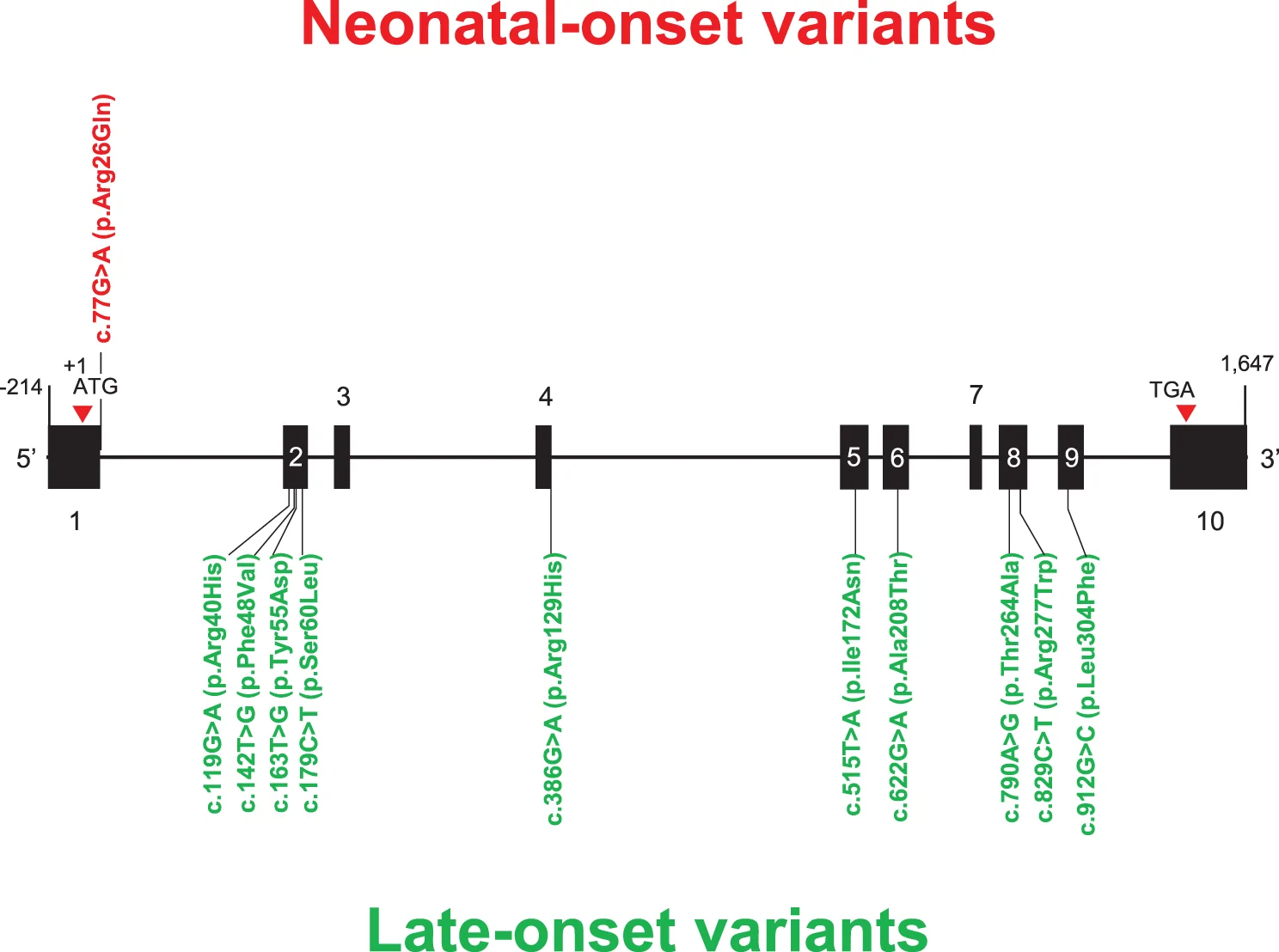
*OTC* gene variants in Neonatal-onset vs. Late-onset types

To further investigate genotype–phenotype correlations in OTCD, we analyzed clinical data from 18 male patients with confirmed pathogenic *OTC* variants (Tables 7, 8, 9, 10). While certain variants were associated with typical neonatal- or late-onset presentations (Fig. 1), clinical severity and symptomatology were not always consistent—even among individuals harboring the same variant.

**Table 7:** The variants and manifestations in adult male patients with OTCD. Neurological manifestations

Patient ID	Nucleic acid	Onset time	Ammonia levels (μmol/L)	Hypoglycemia	Hyperammonemia	Hyperammonemic coma	Seizure	Hypertonia or Hypotonia	Impaired eyesight	Abnormal brain MRI or CT	Abnormal EEG	CP like symptoms
**OTC-1**	**c.77 G > A**	**Neonatal**	**2,349**	-	**〇**	**〇**	**〇**	**〇**	**NA**	**〇**	**〇**	**〇**
**OTC-2**	**c.77 G > A**	**Neonatal**	**212**	-	**-〇**	-	-	-	-	**NA**	**NA**	-
**OTC-4**	**c.119 G > A**	**Late**	**894**	**〇**	**〇**	**〇**	**〇**	**〇**	-	**〇**	**〇**	-
**OTC-5**	**c.119 G > A**	**Late**	**460**	-	**-〇**	-	-	-	-	-	**NA**	-
**OTC-6**	**c.119 G > A**	**Late**	**640**	-	**〇**	**〇**	-	-	-	-	-	-
**OTC-7**	**c.119 G > A**	**Late**	**141**	-	**〇**	**〇**	**〇**	-	-	-	**〇**	-
**OTC-8**	**c.119 G > A**	**Late**	**444**	-	**〇**	**〇**	**〇**	-	-	-	-	-
**OTC-10**	**c.142T > G**	**Late**	**404**	-	**〇**	**NA**	**NA**	**NA**	**NA**	**NA**	**NA**	**NA**
**OTC-11**	**c.163T > G**	**Late (pre)**	**66**	-	**〇**	-	-	-	-	-	-	-
**OTC-12**	**c.179C > T**	**Late**	**NA**	-	**〇**	**〇**	-	-	-	**〇**	-	-
**OTC-14**	**c.386 G > C**	**Late**	**274**	-	**〇**	**〇**	**〇**	-	-	**NA**	**NA**	-
**OTC-15**	**c.515T > A**	**Late**	**274**	-	**〇**	**〇**	-	-	-	-	-	-
**OTC-24**	**c.622 G > A**	**Late**	**483**	-	**〇**	**〇**	-	-	-	-	-	-
**OTC-27**	**c.790A > G**	**Late**	**NA**	-	**〇**	**〇**	-	-	-	-	-	-
**OTC-28**	**c.829C > T**	**Late**	**207**	-	**〇**	**〇**	-	-	-	-	**NA**	-
**OTC-29**	**c.829C > T**	**Late**	**183**	-	**〇**	-	-	-	-	**NA**	**NA**	-
**OTC-31**	**c.912 G > C**	**Late**	**169**	-	**〇**	-	-	-	-	-	-	-

NA: Not available, ID: Intellectual disability, CP: cerebral palsy, EEG: electroencephalogram. 〇: Yes, -: No. Late (pre): This variant is generally considered to be associated with late-onset OTCD, however, the patient carrying this variant was diagnosed prenatally”

**Table 8:** B gastrointestinal manifestations

Patient ID	Nucleic acid	Hepatomegaly	Spleen enlargement	Aversion of protein	Anorexia	Nausea or Vomiting	Intractable diarrhea	Liver damage (abnormal blood test results)	Liver damage (abnormal imaging test results)	LT
**OTC-1**	**c.77 G > A**	**〇**	-	**NA**	-	-	-	**〇**	-	-
**OTC-2**	**c.77 G > A**	-	-	**NA**						**〇**
**OTC-4**	**c.119 G > A**	-	-	**〇**						-
**OTC-5**	**c.119 G > A**	-	-	-						-
**OTC-6**	**c.119 G > A**	-	-	-						-
**OTC-7**	**c.119 G > A**	-	-	**〇**						-
**OTC-8**	**c.119 G > A**	-	-	**NA**						-
**OTC-10**	**c.142T > G**	-	-	**NA**	**NA**	-	-	**〇**	**NA**	**〇**
**OTC-11**	**c.163T > G**	-	-	-	-	**〇**	-	**〇**	**NA**	-
**OTC-12**	**c.179C > T**	-	-	-	-	-	-	-	-	**〇**
**OTC-14**	**c.386 G > C**	**〇**	-	-	**〇**	**〇**	-	-	-	**〇**
**OTC-15**	**c.515T > A**	-	-	-	**〇**	**〇**	**〇**	**〇**	**〇**	-
**OTC-24**	**c.622 G > A**	-	-	-	-	-	-	-	-	-
**OTC-27**	**c.790A > G**	-	-	-	-	**〇**	-	-	-	-
**OTC-28**	**c.829C > T**	-	-	-	-	**〇**	-	-	-	-
**OTC-29**	**c.829C > T**	-	-	**NA**	-	**〇**	-	**〇**	-	-
**OTC-31**	**c.912 G > C**	-	-	**NA**	**〇**	**〇**	-	-	-	**〇**

NA: Not available, LT: Liver transplant

**Table 9:** C hematological manifestations

Patient ID	Nucleic acid	Leukopenia	Anemia	Thrombocytopenia	Bleeding tendency
**OTC-1**	**c.77 G > A**	-	-	-	-
**OTC-2**	**c.77 G > A**	**〇**	**NA**	**NA**	**NA**
**OTC-4**	**c.119 G > A**	-	-	-	-
**OTC-5**	**c.119 G > A**	-	-	-	-
**OTC-6**	**c.119 G > A**	-	-	-	-
**OTC-7**	**c.119 G > A**	**NA**	**NA**	**NA**	**NA**
**OTC-8**	**c.119 G > A**	-	-	-	-
**OTC-10**	**c.142T > G**	-	-	**〇**	-
**OTC-11**	**c.163T > G**	**NA**	**NA**	**NA**	**NA**
**OTC-12**	**c.179C > T**	-	-	-	-
**OTC-14**	**c.386 G > C**	-	-	-	-
**OTC-15**	**c.515T > A**	-	-	-	-
**OTC-24**	**c.622 G > A**	-	-	-	-
**OTC-27**	**c.790A > G**	-	-	-	-
**OTC-28**	**c.829C > T**	**NA**	**NA**	**NA**	**NA**
**OTC-29**	**c.829C > T**	-	**〇**	-	-
**OTC-31**	**c.912 G > C**	-	-	-	-

NA: Not available, ID: Intellectual disability. 〇: Yes, -: No

**Table 10:** D developmental disorders

Patient ID	Nucleic acid	ID	Autism	ADHD	Learning disorder	Adjustment disorder	Communication disorder	Mood disorder	ID	Final academic background
**OTC-1**	**c.77 G > A**	**〇**	**NA**	**NA**	**NA**	**NA**	**NA**	**NA**	**〇**	**Special support school**
**OTC-2**	**c.77 G > A**	-	-	-	-	-	-	-	-	**High school**
**OTC-4**	**c.119 G > A**	**〇**	-	-	**〇**	**〇**	-	**〇**	**〇**	**Bachelor course**
**OTC-5**	**c.119 G > A**	-	-	-	-	-	-	-	-	**Professional training college**
**OTC-6**	**c.119 G > A**	-	-	-	-	-	-	-	-	**Master course**
**OTC-7**	**c.119 G > A**	-	-	-	-	-	-	-	-	**High school**
**OTC-8**	**c.119 G > A**	**〇**	-	-	-	-	-	-	**〇**	**Junior high school**
**OTC-10**	**c.142T > G**	**〇**	**NA**	**NA**	**NA**	**NA**	**NA**	**NA**	**〇**	**Special support school**
**OTC-11**	**c.163T > G**	-	-	-	-	-	-	-	-	**Junior high school**
**OTC-12**	**c.179C > T**	**〇**	-	-	-	-	-	-	**〇**	**Special support school**
**OTC-14**	**c.386 G > C**	-	-	-	-	-	-	-	-	**Master course**
**OTC-15**	**c.515T > A**	**NA**	-	-	-	-	-	-	**NA**	**Junior high school**
**OTC-24**	**c.622 G > A**	-	**NA**	**NA**	**NA**	**NA**	**NA**	**NA**	-	**High school**
**OTC-27**	**c.790A > G**	**〇**	-	-	-	-	-	-	**〇**	**NA**
**OTC-28**	**c.829C > T**	-	-	-	-	-	-	-	-	**Bachelor course**
**OTC-29**	**c.829C > T**	-	-	-	-	-	-	-	-	**NA**
**OTC-31**	**c.912 G > C**	-	-	-	-	**〇**	**〇**	-	-	**Bachelor course**

ADHD: Attention-deficit/hyperactivity disorder, NA: not available. 〇: Yes, -: No

## Discussion

This study characterized the genetic variants and clinical outcomes of adult long-term survivors with UCDs in Japan. As expected, the majority of participants had late-onset disease, reflecting the increased likelihood of long-term survival in this subgroup. In particular, the pathogenic variants identified in male patients with late-onset OTCD were consistent with previously reported late-onset–type mutations [12, 14].

OTCD is an X-linked disorder, while CPS1D, ASS1D, ASLD, ARG1D, and HHH syndrome follow autosomal recessive inheritance patterns. According to the ClinVar database as of July 2025, the number of registered pathogenic variants for OTCD, CPS1D, ASS1D, ASLD, ARG1D, and HHH syndrome were 1,092; 2,097; 904; 928; 582; and 524, respectively. The *OTC* gene is located on chromosome Xp21.1, and the disorder predominantly affects hemizygous males. However, approximately 20% of heterozygous female carriers also exhibit clinical manifestations [26].

In male OTCD patients, disease severity is often determined by variant-specific effects on OTC enzyme structure and function [27, 28]. We have previously shown that the type of pathogenic variant correlates with age of onset and clinical severity, with neonatal-onset cases typically presenting with markedly elevated plasma ammonia levels and severe neurologic outcomes [12, 14].

In contrast, establishing clear genotype–phenotype correlations in autosomal recessive UCDs such as CPS1D, ASS1D, ASLD, ARG1D, and HHH syndrome is more challenging, due to the predominance of compound heterozygous variants. Furthermore, environmental stressors—such as infection, fasting, high protein intake, surgery, or childbirth—can precipitate hyperammonemic crises even in individuals harboring milder or late-onset variants, resulting in clinical features that closely resemble those of neonatal-onset disease (Table 1).

Among OTCD patients harboring the c.77 G > A and c.119 G > A variants, peak plasma ammonia levels varied considerably. Moreover, clinical phenotypes differed even among patients with identical genotypes, underscoring the phenotypic variability of UCDs. While genetic information is useful for predicting age of onset and disease severity—particularly in neonatal-onset cases—it is not sufficient to fully predict long-term clinical outcomes.

Neonatal-onset UCDs are typically associated with severe hyperammonemia, often exceeding 360 μmol/L at presentation [29]. This degree of hyperammonemia is strongly correlated with the risk of irreversible brain injury and adverse neurodevelopmental outcomes [9, 10, 30]. In OTCD-affected families, both male and female offspring may inherit the disorder. If a pathogenic *OTC* variant is identified through prenatal genetic testing, prompt postnatal intervention can significantly reduce the risk of hyperammonemia and improve long-term neurocognitive outcomes, as demonstrated in patient OTC-11 (Tables 1 and 2).

This study has several limitations. First, it was based on a retrospective review of medical records. Second, patients diagnosed during childhood may have been lost to follow-up after transitioning to adult care, resulting in the potential underrepresentation of individuals with poor outcomes. Consequently, our cohort may be biased toward patients with relatively favorable long-term prognoses who remained under continuous medical follow-up.

Third, the present study combined data from two nationwide surveys conducted during different periods (January 2000 to March 2018 and January 2010 to December 2022), involving different patient cohorts and participating institutions. Moreover, cognitive and other clinical outcomes were assessed as part of routine clinical practice rather than according to a standardized study protocol. Because these assessments were performed by both pediatricians and adult physicians with different clinical perspectives, subtle neurodevelopmental, cognitive, or functional impairments may have been evaluated inconsistently across institutions, potentially leading to underestimation of some clinical manifestations.

Fourth, molecular genetic and clinical outcome data were not available for all patients. Although 116 adults with UCDs were identified through the nationwide surveys, only 48 had both molecular genetic confirmation and sufficiently detailed clinical outcome data for genotype–phenotype analysis. In addition, the number of patients with several UCD subtypes, particularly CPS1D, ARG1D, ASLD, and HHH syndrome, was limited. Therefore, subtype-specific observations should be interpreted with caution. Furthermore, our findings may not be generalizable to all Japanese adults with UCDs.

To overcome these limitations and facilitate future prospective studies, the recent establishment of a nationwide UCD registry in Japan represents an important advance. This registry will enable the systematic collection of standardized clinical and genetic data, harmonize outcome assessments across institutions, and improve long-term monitoring of patients with UCDs.

## Conclusion

In this nationwide study, we characterized the clinical and genetic features of adult patients with UCDs in Japan. We identified major pathogenic variants and evaluated their associations with age at onset, clinical severity, and long-term outcomes. Our findings suggest that genotype information may help predict disease onset, clinical severity, and long-term prognosis in patients with UCDs. Further large-scale prospective studies using this nationwide registry are warranted to validate these genotype–phenotype correlations and to identify genetic factors associated with long-term clinical outcomes.

## Electronic supplementary material

Below is the link to the electronic supplementary material.

Supplementary Material 1

Supplementary Material 2

## Data Availability

Data will be made available on request.
