# Supplementary material for: Genotypic and clinical characteristics of long-term surviving adult patients with urea cycle disorders in Japan
Source: Orphanet J Rare Dis. 2026 Jul 31;21:380. doi: 10.1186/s13023-026-04524-x (PMC13715130; doi:10.1186/s13023-026-04524-x)
Supplement: Supplementary file 1 [file 13023_2026_4524_MOESM1_ESM.docx]

**Supplementary Data 1.** The relation between blood ammonia levels and intellectual levels in patients with UCDs

A. UCDs

| UCDs | Blood ammonia ≥ 360 | Blood ammonia < 360 | Total |
| --- | --- | --- | --- |
| Intellectual disability | 11 | 8 | 19 |
| No intellectual disability | 7 | 18 | 25 |
| Total | 18 | 26 | 44 |

*P* = 0.046*

B. OTCD

| OTCD | Blood ammonia ≥ 360 | Blood ammonia < 360 | Total |
| --- | --- | --- | --- |
| Intellectual disability | 6 | 2 | 8 |
| No intellectual disability | 6 | 16 | 22 |
| Total | 12 | 18 | 30 |

*P* = 0.018*
