# Supplementary material for: Genotypic and clinical characteristics of long-term surviving adult patients with urea cycle disorders in Japan
Source: Orphanet J Rare Dis. 2026 Jul 31;21:380. doi: 10.1186/s13023-026-04524-x (PMC13715130; doi:10.1186/s13023-026-04524-x)
Supplement: Supplementary file 2 [file 13023_2026_4524_MOESM2_ESM.docx]

Supplementary Data 2. The variants and manifestations in adult patients with OTCD

| Variant type | Nucleic acid | Amino acid | Onset time | Patient number (ID) | Ammonia levels  (μmol/L) | Hyperammonemic coma |
| --- | --- | --- | --- | --- | --- | --- |
| Missense | c.77G>A | p.Arg26Gln | Neonatal | **2 (OTC-1, 2)** | 2,349, 212 | Y, N |
|  | c.119G>A | p.Arg40His | Late | **5 (OTC-4-8)** | 894, 460, 640, 141, 444 | Y, N, Y, Y, Y |
|  |  |  |  | 1 (OTC-3) | 402 | N |
|  | c.140A>T | p.Asp47Ile | Late | 1 (OTC-9) | 90 | Y |
|  | c.142T>G | p.Phe48Val | Late | **1 (OTC-10)** | 404 | NA |
|  | c.163T>G | p.Tyr55Asp | Late (pre) | **1 (OTC-11)** | 66 | N |
|  | c.179C>T | p.Ser60Leu | Late | **1 (OTC-12)** | NA | Y |
|  | c.254T>A | p.Ile85Asn | Late | 1 (OTC-13) | 119 | Y |
|  | c.386G>C | p.Arg129His | Late | **1 (OTC-14)** | 274 | Y |
|  | c.515T>A | p.Ile172Asn | Late | **1 (OTC-15)** | 274 | Y |
|  | c.533C>T | p.Thr178Met | Late | 2 (OTC-18, 19) | 280, 116 | Y, Y, Y, Y |
|  | c.542A>G | p.Glu181Gly | Late | 1 (OTC-20) | 424 | Y |
|  | c.583G>A | p.Gly195Arg | Late | 1 (OTC-21) | 1,174 | Y |
|  | c.604C>T | p.His202Tyr | Late | 1 (OTC-22) | 344 | Y |
|  | c.617T>G | p.Met206Arg | Late | 1 (OTC-23) | NA | NA |
|  | c.622G>A | p.Ala208Thr | Late (dead at 46y9m) | **1 (OTC-24)** | 483 | Y |
|  | c.626C>T | p.Ala209Val | Late | 1 (OTC-25) | 117 | N |
|  | c.643C>T | p.Leu215Phe | Late | 1 (OTC-26) | 180 | Y |
|  | c.790A>G | p.Thr264Ala | Late | **1 (OTC-27)** | NA | Y |
|  | c.829C>T | p.Arg277Trp | Late | **2 (OTC-28, 29)** | 207, 183 | Y, N |
|  | c.912G>C | p.Leu304Phe | Late | **1 (OTC-31)** | 169 | N |
|  | c.959G>T | p.Arg320Leu | Late (dead at 61y10m) | 1 (OTC-33) | 1,115 | Y |
| Frame-shift | c.523_536del | p.Asp175Profs*5 | Late | 2 (OTC-16, 17) | 233, 280 | N, N |
| Nonsense | c.894G>A | p.Trp298* | Late | 1 (OTC-30) | 570 | N |
|  | c.940G>T | p.Glu314* | Late | 1 (OTC-32) | 166 | Y |
| Large-rearrangement | Ex1-6dup | - | Late | 1 (OTC-34) | 129 | N |

Late (pre): diagnosed prenatally

**Bold**: male, NA: not available, Y: Yes, N: No
